# Efficient transdermal delivery of functional protein cargoes by a hydrophobic peptide MTD 1067

**DOI:** 10.1038/s41598-022-14463-9

**Published:** 2022-06-27

**Authors:** Hee Je Shin, Sun Uk Bak, Ha Na La, Jin Sun Kang, Hwa Hyun Lee, Hyo Jung Eom, Byung Kyu Lee, Hyun Ah Kang

**Affiliations:** 1 ProCell R&D Center, ProcellTherapeutics, Inc., #1009 Ace-Twin Tower II, 273, Digital-ro, Guro-gu, Seoul, 08381 Republic of Korea; 2 Department of Life Science, College of Natural Science, Chung-Ang University, 84, Heukseok-ro, Dongjak-gu, Seoul, 06974 Republic of Korea

**Keywords:** Biomaterials - proteins, Protein delivery, Drug delivery

## Abstract

The skin has a protective barrier against the external environment, making the transdermal delivery of active macromolecules very difficult. Cell-penetrating peptides (CPPs) have been accepted as useful delivery tools owing to their high transduction efficiency and low cytotoxicity. In this study, we evaluated the hydrophobic peptide, macromolecule transduction domain 1067 (MTD 1067) as a CPP for the transdermal delivery of protein cargoes of various sizes, including growth hormone-releasing hexapeptide-6 (GHRP-6), a truncated form of insulin-like growth factor-I (des(1-3)IGF-I), and platelet-derived growth factor BB (PDGF-BB). The MTD 1067-conjugated GHRP-6 (MTD-GHRP-6) was chemically synthesized, whereas the MTD 1067-conjugated des(1-3)IGF-I and PDGF-BB proteins (MTD-des(1-3)IGF-I and MTD-PDGF-BB) were generated as recombinant proteins. All the MTD 1067-conjugated cargoes exhibited biological activities identical or improved when compared to those of the original cargoes. The analysis of confocal microscopy images showed that MTD-GHRP-6, MTD-des(1-3)IGF-I, and MTD-PDGF-BB were detected at 4.4-, 18.8-, and 32.9-times higher levels in the dermis, respectively, compared to the control group without MTD. Furthermore, the MTD 1067-conjugated cargoes did not show cytotoxicity. Altogether, our data demonstrate the potential of MTD 1067 conjugation in developing functional macromolecules for cosmetics and drugs with enhanced transdermal permeability.

## Introduction

Cell penetrating peptide (CPP) is a peptide composed of 5–30 amino acids that can penetrate cell membranes. They can transport a variety of active substances into the cells by translocating them across the plasma membrane. Since the Tat protein of human immunodeficiency virus type I (HIV-1), consisting of 86 amino acids, was demonstrated to be capable of intracellular internalization in 1988^1^, several CPPs have been identified and studied, including homeodomain-derived peptides^2^, amphipathic peptides^3,4^, synthetic and/or chimeric cell-penetrating peptides^5^, and signal sequence-based peptides^6,7^. CPPs have been applied in a variety of industrial fields because of their special ability to transport various cargoes into cells or the skin. CPPs are considered powerful tools for biological studies, drug development, and transdermal delivery for cosmetics. For instance, CPPs have been used for efficient delivery of contrast agents for cell imaging purposes, such as quantum dots^8^ or metal chelates^9^. CPPs have been exploited for intracellular delivery of various nucleic acids, including oligomers, plasmid DNA^10^, siRNA^11^, and DNA^12,13^. They have also been applied as delivery systems for several drugs, ranging from nanoparticles to therapeutic proteins^14–16^.

The skin, as the body's largest organ, is an important tissue for drug administration and cosmetic applications because the delivery of active ingredients, such as pharmaceuticals and functional materials through the skin, is simple and provides many benefits to patients and customers. However, the skin has a protective barrier against the external environment; therefore, the task of skin penetration remains challenging in the development of active ingredients for advanced pharmaceutical and functional cosmetics, largely because of problems associated with low permeation efficiency^17,18^. Nevertheless, several challenges have been addressed to overcome this issue. CPPs are considered promising carriers for the transdermal delivery of macromolecules across skin barriers as well as biological membranes. Typically, conventional CPPs contain a large proportion of positively charged amino acids, especially arginine, or an alternating sequence of charged and nonpolar residues^19–21^. Despite their popularity as cellular delivery tools, systematic delivery of macromolecules using CPPs with cationic protein transduction domains has proven difficult because of the sequestration of significant amounts of proteins into membrane-bound and endosomal compartments. Macromolecular intracellular transduction technology (MITT) was developed as a novel cell penetration technology that exploits the macromolecule transduction domain (MTD), a hydrophobic peptide with electrostatic properties different from those of CPPs composed of charged amino acids^22^. Hydrophobic MTDs promote the bidirectional transfer of peptides and proteins across the plasma, thus improving the intracellular delivery efficiency of cargoes upon conjugation^23^. The first reported MTD peptides were cell-permeable synthetic peptides derived from the membrane translocation sequence (MTS) of the Kaposi fibroblast growth factor (FGF-4) signal peptides^24^. Recombinant Cre recombinase fusion proteins bearing the 12 amino acid MTS from FGF-4 have been constructed to transduce enzymatically active Cre proteins directly into mammalian cells^25^. Various synthetic MTDs were subsequently designed to assign cell membrane permeability by linking them to several macromolecules, such as proteins or nucleic acids that are difficult to penetrate through cell membranes^22,26–29^. MTDs have shown advantageous properties, such as effective cell penetration rate and cell-to-cell delivery within tissues, supporting their high potential in the development of therapeutic substances and cosmetics that require transdermal permeability and transferability of active substances.

MTD 1067 was identified in a library of modified MTDs that were originally synthesized based on the signal sequences of secretory or membrane proteins^30^. In recent, peptides and proteins that are chemically synthesized or produced as recombinant proteins have drawn attention as functional components in the cosmeceutical field, and many of them have excellent nontoxicity and stability records^31,32^. In this study, we evaluated whether transdermal permeability can be efficiently conferred by applying MTD 1067 to protein cargoes of various sizes. For this, MTD 1067 was fused to 0.8 kDa growth hormone-releasing hexapeptide-6 (GHRP-6), 7.7 kDa truncated form of insulin-like growth factor-I (des(1-3)IGF-I), and platelet-derived growth factor BB (PDGF-BB), a dimeric protein of 24.8 kDa. The biological activity, cytotoxicity, and transdermal delivery efficiency of the three MTD 1067-conjugated protein cargoes were assessed, demonstrating that transdermal permeation functionality could be imparted without changing the intrinsic activity of cargoes by MTD 1067 conjugation.

## Results

### MTD 1067-conjugated peptides and recombinant proteins

**Figure 1:**
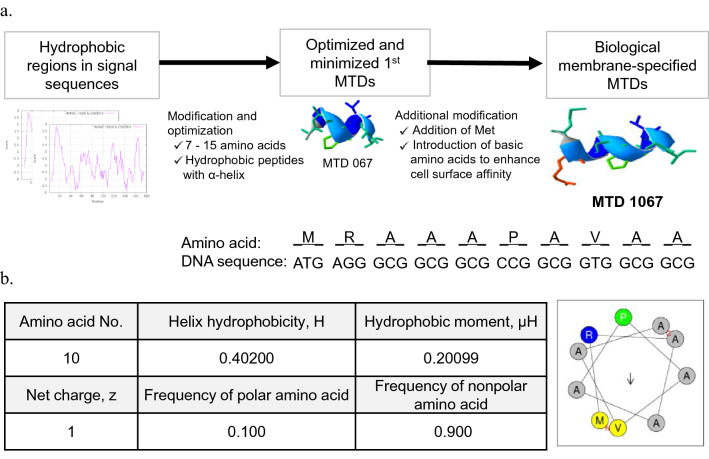
Physicochemical properties of MTD 1067. (**a**) Optimization and modification of synthetic MTD peptides designed based on hydrophobic regions in signal peptide sequences. MTD 1067 was modified from one of the first-generation MTDs, MTD 067 (AAAPAVAA) to increase biological membrane accessibility and permeability^30‚58^. The first generation of synthetic MTDs was developed by modifying the hydrophobic region in the signal sequences of various proteins to contain non-polar amino acids, such as alanine, valine, leucine, isoleucine, and proline, in different combinations to increase affinity with the hydrophobic lipid bilayer of the intracellular plasma membrane. Subsequently, positively charged hydrophilic amino acids, such as arginine, histidine, and lysine, were additionally introduced into the conventional hydrophobic MTDs to increase the accessibility of peptides to the negatively charged surface of the cell membrane. The N-terminal was also modified to contain methionine, which is the universal start codon in the signal peptide. The secondary structure of MTD 1067 was predicted using a de novo peptide structure prediction tool, PEP-FOLD3. (**b**) Alpha helix structure and physicochemical properties of MTD 1067. The helix wheel property and secondary structure of MTD 1067 were analyzed using HELIQUEST (http://heliquest.ipmc.cnrs.fr/)^59^ and PEP-FOLD 3.5 (https://mobyle.rpbs.univ-paris-diderot.fr/)^60–62^, respectively.

The hydrophobic peptide MTD 1067, evaluated in this study, is the optimized version of MTD 067 (AAAPAVAA), which was initially derived from the signal sequence of the hypothetical protein SCO2321 in *Streptomyces coelicolor* A3(2) (NCBI reference sequence: WP_003976492.1). The MTD 1067 consists of ten amino acids (MRAAAPAVAA) and has 90% hydrophobic amino acid and alpha-helical properties (Fig. 1). For conjugation with MTD 1067, peptide and protein cargoes with different molecular weights and biochemical properties, GHRP-6, des(1-3)IGF-I, and PDGF-BB, were selected. The cargoes selected for MTD 1067 conjugation possess several biological activities useful to be developed as pharmaceutical and cosmeceutical components (Table 1).

**Table 1:** Characteristics of the cargoes selected for MTD conjugation.

Cargo	Gene and peptide accession numbers	Molecular weight, Da	Functional structure	Biological activity	Refs.
Growth hormone-releasing peptide-6 (GHRP-6)	Pubchem SID: 135,652,165 PubChem CID: 4,345,065	873	Peptide	Antagonist of α-MSH Collagen synthesis	^39,40^
MTD-GHRP-6	Synthetic peptide	1898			
Truncated form of insulin-like growth factor-I (des(1-3)IGF-I)	BankIt2569064 Des (1-3)IGF-I ON156849	7785	Monomeric protein	Cell growth and maintenance Hair growth	^43,44^
MTD-des(1-3)IGF-I	BankIt2569065 MTD-des(1-3)IGF-I ON156850	8564			
Platelet-derived growth factor (PDGF) BB	BankIt2569068 PDGFB ON156851	24,851	Dimeric protein	Cell growth and division Production of matrix proteins	^52,47^
MTD-PDGF-BB	BankIt2569071 MTD-PDGFB ON156852	26,671			

**Figure 2:**
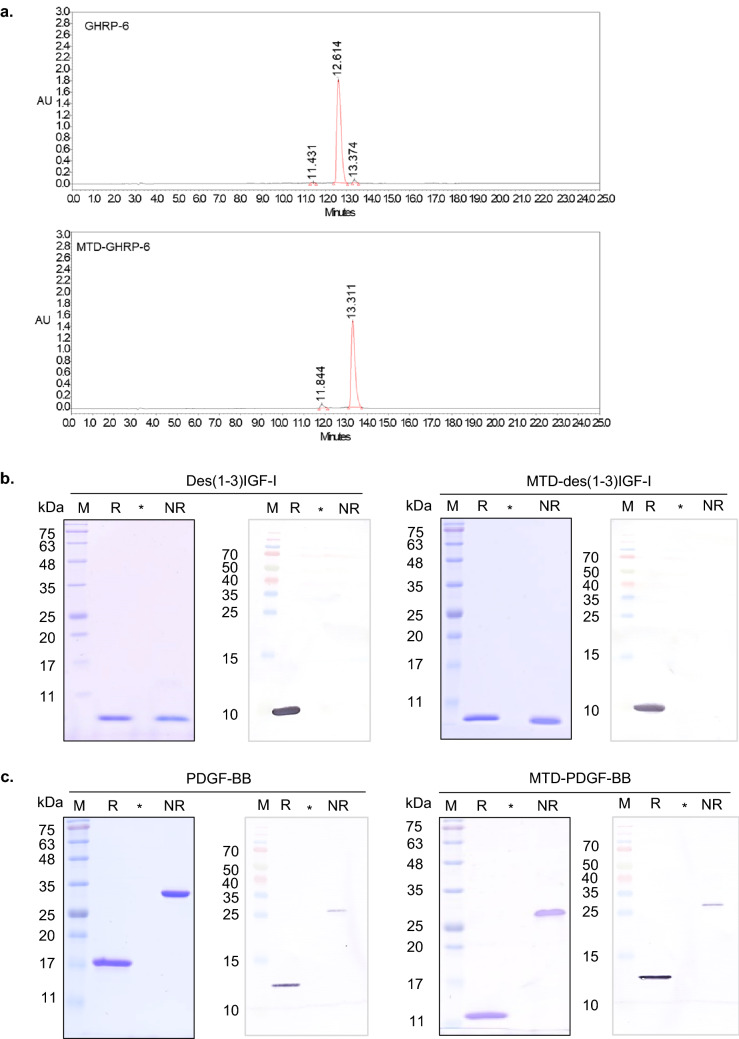
Purification of peptide and recombinant proteins. (**a**) RP-HPLC analysis of purified GHRP-6 and MTD-GHRP-6 peptides using the 20–60% solvent B gradient method for 25 min. (**b**) SDS-PAGE and western blot analysis of des(1-3)IGF-I and MTD-des(1-3)IGF-I proteins, (**c**) SDS-PAGE and western blot analysis of PDGF-BB and MTD-PDGF-BB proteins. M, molecular weight marker; R, reduced form; NR, non-reduced form; *, blank lane without sample loading. The raw data of western blot analysis in (**b**) and (**c**), respectively, were presented in Supplementary Information as Fig. S4a,b.

The peptides GHRP-6 and MTD-GHRP-6 were chemically synthesized using the SPPS-peptide synthesis method and purified by phase column chromatography (Supplementary Fig. S1a). The purity and identity of the final lyophilized peptides were analyzed using HPLC (Fig. 2a) and mass spectrometry (Supplementary Fig. S3). The purities of GHRP-6 and MTD-GHRP-6 were 97.67% and 96.55%, respectively, and the molecular weights of the final synthesized peptides were 873.3 Da and 1898.7 Da, respectively, which were consistent with the theoretical values (Table 1). The recombinant des(1-3)IGF-I and MTD-des(1-3)IGF-I proteins, expressed as insoluble proteins in *E. coli*, were purified after refolding through a continuous purification process, including SP-Sepharose and size exclusion chromatography (Supplementary Fig. S1b). The purities of des(1-3)IGF-I and MTD-des(1-3)IGF-I were confirmed to be 99.5% and 99.1%, respectively. Western blot analysis using a monoclonal antibody against des(1-3)IGF-I was conducted to confirm that the purified protein was a recombinant human des(1-3)IGF-I (Fig. 2b). The des(1-3)IGF-I and MTD-des(1-3)IGF-I protein bands were evidently detected in the reduced condition (R) but not detectable in the non-reduced condition (NR) in the western blot. This might be partly due to the reduced binding affinity to the monoclonal antibody by partial conformational changes generated by the deletion of three amino acids at the N-terminus of IGF-I.

The recombinant PDGF-BB and MTD-PDGF-BB proteins, expressed as insoluble proteins in *E. coli*, were purified through refolding and purification using CM-Sepharose chromatography (Supplementary Fig. S1c). The purity of PDGF-BB and MTD-PDGF-BB was confirmed to be 99.5% and 99.1%, respectively, by SDS-PAGE analysis. Using western blotting, the purified protein was further validated as recombinant human PDGF-BB (Fig. 2c). The PDGF-BB and MTD-PDGF-BB protein bands were more evidently detected in the reduced condition (R) than in the non-reduced condition (NR) in the western blot, indicating that some epitopes of PDGF-BB in dimer form might be less accessible to the anti-human PDGF-BB polyclonal antibody used in this study.

### Biological activities of MTD 1067-fused peptide and recombinant proteins

**Figure 3:**
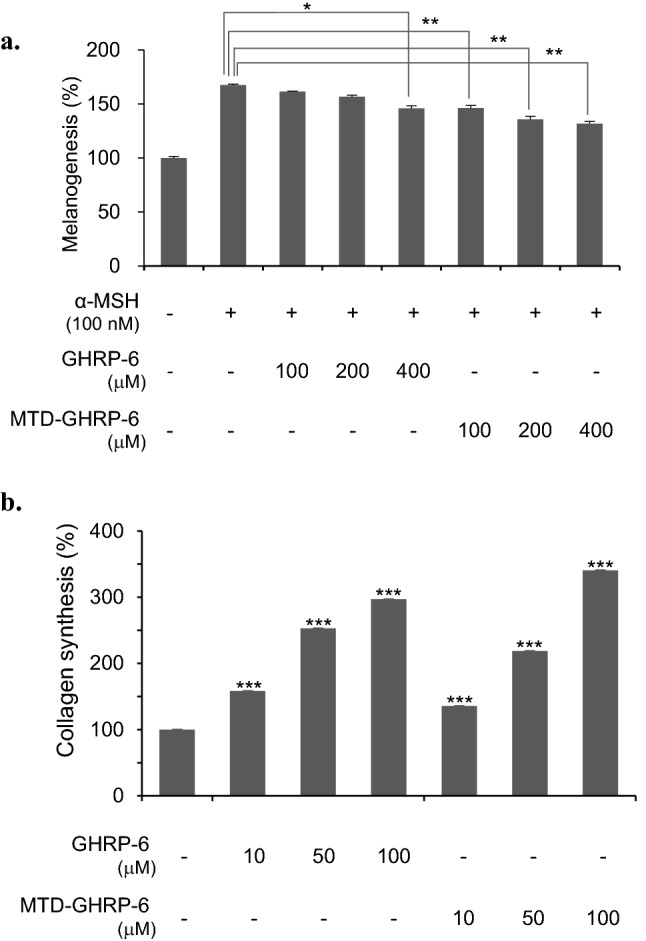
Biological activity of the MTD 1067-conjugated peptide. (**a**) Melanogenesis inhibition activity of GHRP-6 and MTD-GHRP-6 using murine melanoma cell line B16F10. α-MSH, α-melanocyte stimulating hormone. The cell culture treated with α-MSH only in the absence of GHRP-6 and MTD-GHRP-6 was used as the control to calculate the melanogenesis activity (%). Asterisks indicate statistical significance determined by Student's t-test. Anti-melanogenesis activity was confirmed by statistical significance compared the α-MSH-treated cells without the GHRP-6 peptides. (**b**) Collagen synthesis effect of GHRP-6 and MTD-GHRP-6. The amount of collagen in the HDFa culture supernatant was measured using a procollagen type I C-peptide (PIP) EIA kit. The collagen synthesis effect was confirmed with statistical significance by comparison with the cells cultivated without GHRP-6 and MTD-GHRP-6. Asterisks indicate statistical significance determined by Student's t-test. (*p < 0.05, **p < 0.01, ***p < 0.001).

The biological activity of the MTD-GHRP-6 peptide was measured by focusing on activities that can be utilized for the development of cosmeceutical products^33^. GHRP-6 is known as an antagonist of α-melanocyte stimulating hormone (α-MSH), which induces the activation of melanocytes (Table 1). Both GHRP-6 and MTD-GHRP-6 peptides showed anti-melanogenic activity in a concentration-dependent manner with the slightly improved activity of MTD-GHRP-6 (Fig. 3a). Increased accumulation of collagen by MTD-GHRP-6 was also observed in a concentration-dependent manner from 10 to 100 μM, similar to GHRP-6 (Fig. 3b).

**Figure 4:**
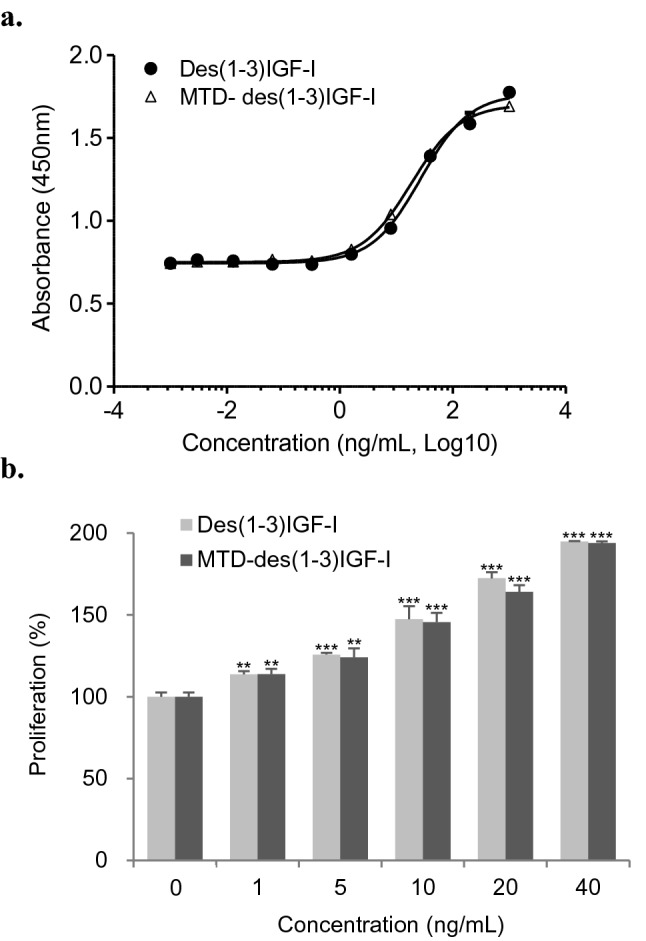
Cell proliferation activity of des(1-3)IGF-I and MTD-des(1-3)IGF-I in FDC-P1 cells (**a**) and HaCaT cells (**b**). Cell proliferation activity in FDC-P1 cells was analyzed by measuring the color development using WST-1 and in HaCaT cells by crystal violet staining. The results were expressed as % compared to the cell culture without treatment of des(1-3)IGF-I and MTD-des(1-3)IGF-I. Asterisks indicate statistical significance determined by Student's t-test. (*p < 0.05, **p < 0.01, ***p < 0.001).

The cell proliferation activities of des(1-3)IGF-I and MTD-des(1-3)IGF-I were evaluated using the interleukin-3 (IL-3)-dependent cell line FDC-P1, which is a general method for measuring the biological activity of IGF1^34^. The purified des(1-3)IGF-I and MTD-des(1-3)IGF-I proteins enhanced the growth of hematopoietic cells in a concentration-dependent manner, with a 50% effective dose (ED_50_) of des(1-3)IGF-I and MTD-des(1-3)IGF-I as 27 ng/mL and 18 ng/mL, respectively. Both des(1-3)IGF-I and MTD-des(1-3)IGF-I also showed almost identical cell proliferation activity in the analysis using HaCaT cells, a keratinocyte cell line from adult human skin (Fig. 4a,b). MTD-des(1-3)IGF-I protein was confirmed to have an identical cell proliferation effect as des(1-3)IGF-I.

**Figure 5:**
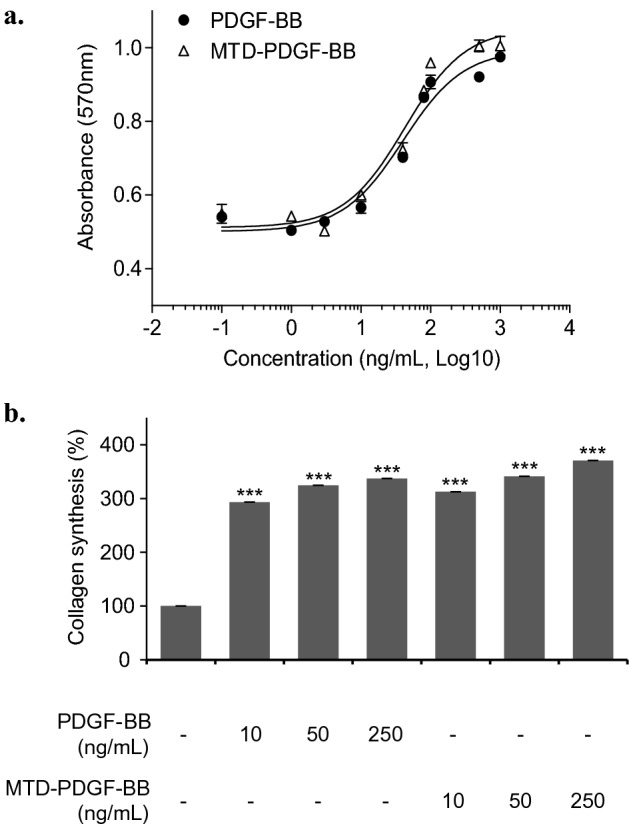
Biological activity of PDGF-BB and MTD-PDGF-BB. (**a**) Cell proliferation activity of PDGF-BB and MTD-PDGF-BB using fibroblast cell line NIH 3T3 cell. Cell proliferation activity in NIH 3T3 cells was analyzed by measuring the color development using MTT. All data are expressed as mean ± standard deviation based on triplicates of at least two independent experiments. (**b**) Collagen synthesis effect of PDGF-BB and MTD-PDGF-BB. The amount of collagen in the HDFa culture supernatant was measured using a procollagen type I C-peptide (PIP) EIA kit. Data are expressed as mean ± S.E. based on triplicates of at least two independent experiments. Asterisks indicate statistical significance compared with the cell culture without treatment of PDGF-BB and MTD-PDGF-BB, as determined by the student's t-test (*p < 0.05, **p < 0.01, ***p < 0.001).

The cell proliferation assay with PDGF-BB and MTD-PDGF-BB using the fibroblast cell line NIH 3T3 revealed that the ED_50_ of PDGF-BB and MTD-PDGF-BB was 42 ng/mL and 40 ng/mL, respectively (Fig. 5a). The biological activity of PDGF-BB in a dimer showed a similar activity pattern, regardless of MTD 1067 conjugation, but the MTD 1067-conjugated PDGF-BB exhibited a little bit increased cell proliferation activity. It was also shown that MTD-PDGF-BB had a similar level of collagen synthesis efficacy in a concentration-dependent manner, as shown in the control PDGF-BB protein (Fig. 5b). However, at higher concentrations, the enhancing activity on collagen synthesis was more apparent with MTD-PDGF-BB. When we compared statistically the protein cargoes with and without MTD 1067 conjugation, the increased biological activities of MTD-PDGF-BB compared to the original PDGF-BB was shown to be statistically significant, particularly at high concentrations. Altogether, these results confirmed that the intrinsic activity of PDGF-BB was not affected but rather improved by MTD 1067 conjugation. The MTD-PDGF-BB protein in dimer form have an improved concentration-dependent efficacy in cell proliferation and collagen synthesis assays on fibroblasts compared to PDGF-BB.

### Skin penetration activity and cytotoxicity of MTD 1067-conjugated peptide and recombinant proteins

**Figure 6:**
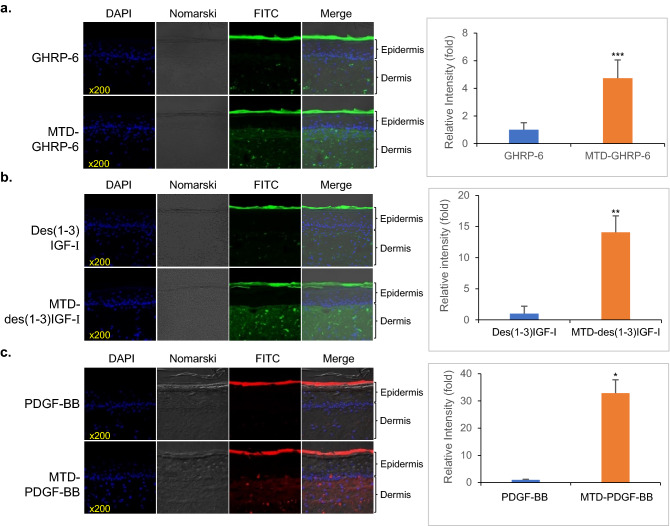
Transdermal delivery of MTD 1067-conjugated peptide and recombinant proteins in artificial skin model. Comparison of skin penetration (left) and permeability analysis using fluorescence intensity on the confocal image (right) were made with (**a**) FITC-labeled GHRP-6 and MTD-GHRP-6, (**b**) FITC-labeled des(1-3)IGF-I and MTD-des(1-3)IGF-I, (**c**) Rhodamine-labeled PDGF-BB and MTD-PDGF-BB. Each of the fluorescence intensity values per equivalent area are expressed as mean ± S.E. based on analysis of at least three independent images. Asterisks indicate statistical significance compared with the cargo control, as determined by the student's t-test (*p < 0.05, **p < 0.01, ***p < 0.001).

To evaluate the skin penetration activity, FITC- or rhodamine-labeled cargoes were applied to the artificial skin for 24 h, and the test tissue was cut into sections for the analysis of confocal images. The MTD-GHRP-6 peptide labeled with FITC showed a higher fluorescence intensity in the dermal layer than the original GHRP-6 peptide. Comparative analysis of FITC-peptide uptake, based on the fluorescence intensity in the equivalent area of each confocal image, confirmed that the permeability of FITC-labeled MTD-GHRP-6 was increased by approximately five times that of GHRP-6 (Fig. 6a). In the case of FITC-labeled des(1-3)IGF-I and MTD-des(1-3)IGF-I, MTD-des(1-3)IGF-I showed 14 times higher fluorescence intensity in the dermal layer than des(1-3)IGF-I (Fig. 6b). The rhodamine-labeled MTD-PDGF-BB also showed higher fluorescence intensity in the dermal layer, with an improvement of approximately 32 times compared to PDGF-BB (Fig. 6c).

**Figure. 7:**
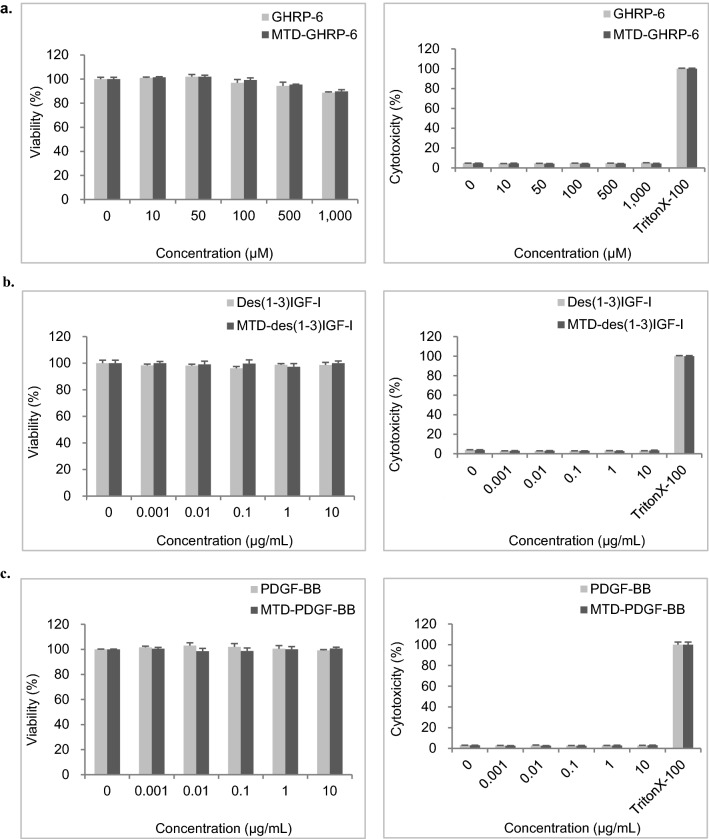
Cell viability analysis using MTT assay (left panels) and cytotoxicity analysis using LDH assay (right panels) in HDFa cell. (**a**) GHRP-6 and MTD-GHRP-6, (**b**) des(1-3)IGF-I and MTD-des(1-3)IGF-I, (**c**) PDGF-BB and MTD-PDGF-BB. The μM concentrations correspond to 0, 8.7, 43.7, 87.3, 436.5 and 873 μg/mL for GHRP-6 and 0, 19.0, 94.9, 189.8, 949 and 1898 μg/mL for MTD-GHRP-6, respectively. Data are expressed as mean ± S.E. based on triplicates of at least two independent experiments. The results were expressed as % compared to the cell culture without treatment of the purified peptide or proteins.

To examine the cytotoxicity of MTD 1067-conjugated peptides and proteins, primary human dermal fibroblasts (HDFa) were treated with GHRP-6 peptides in range of 0, 10, 50, 100, 500, and 1000 μM and recombinant proteins in a range of 0, 0.001, 0.01, 0.1, 1, and 10 μg/mL. After 24 h of incubation, cell viability and cytotoxicity were measured using the MTT and LDH activity assays, respectively. HDFa cells only, without cargo, were treated with Triton-×100 and used as an LDH positive control, which was set to 100% cell lysis. Comparison of cell viability (Fig. 7, left panels) and LDH values (Fig. 7, right panels) of HDFa cells incubated with peptide or protein cargoes showed that there was no cytotoxicity up to 10 µg/mL in all tested groups. This strongly suggested that MTD 1067 conjugation is a safe approach without cytotoxicity.

## Discussion

Skin penetration is a challenging issue in the development of active ingredients for use in advanced pharmaceutical and functional cosmetics. CPPs, also known as protein transduction domains (PTDs) or membrane-translocating sequences (MTSs), have been accepted as useful delivery tools because of their high transduction efficiency and low cytotoxicity^35^. CPPs can be classified based on their physicochemical properties, such as the net charge of peptides and the number of charged amino acids, thus being divided into three classes: cationic, amphipathic, and hydrophobic^3^. CPP can also be divided into anionic, cationic, and hydrophobic based on charged groups,hydrophilic and hydrophobic depending on solubility; and cyclized or stapled form, alpha helix, and random coil by secondary structure^36^. The intracellular transduction mechanisms of CPPs have not yet been elucidated clearly at the molecular interaction level; however, in general, cationic CPPs are considered to exploit the endocytosis pathway as a penetrating mechanism^19,20^, whereas amphipathic CPPs use endocytosis and/or direct penetration pathways to achieve translocation into cells^21,37^. Hydrophobic CPPs have been proposed to be delivered intracellularly through a direct penetrating pathway without energy consumption during the cell internalization process, which might be advantageous for cell-to-cell delivery^27^. In this study, we evaluated the capacity of the hydrophobic peptide MTD 1067 for transdermal delivery of protein cargoes, which is a critical property for developing functional ingredients useful for drugs and cosmetics with improved skin permeability.

The hydrophobic peptide MTD 1067, composed of ten amino acids (MRAAAPAVAA), is an optimized synthetic MTD that was initially reconstituted based on the signal peptide sequence of the hypothetical protein SCO2321 in *Streptomyces coelicolor* A3(2). MTD 1067 was further modified from the first synthetic MTD generated by the addition of N-terminal Met and one basic amino acid at the second position to increase accessibility to the negatively charged surface molecules of the cell membrane (Fig. 1). FITC-conjugated MTD 1067 showed more efficient epithelial penetration into the deep tissue of the stratum corneum of the skin in the epithelial tissue of female ICR mice compared to FITC-conjugated Tat used as a control^38^. Conjugation of MTD 1067 to coumaric acid and acetylated pentapeptide (Lys Thr Thr Lys Ser) retained the activity of tyrosinase inhibitor and collagenase, respectively, suggesting that MTD-coumaric acid and pentapeptide may be used as active ingredients in cosmetics^38^. MTD 1067 has some noticeable properties: it contains six alanine residues carrying a small side chain, one central proline to increase flexibility, and one basic amino acid residue, arginine, in the second position after N-terminal methionine. The hydrophobic moment value indicates that MTD 1067 has an α-helical structure that is typically present in membrane proteins. These properties suggest that MTD 1067 may have an affinity for epidermal and dermal components composed of fatty acids and various hydrophobic molecules, thereby contributing to transdermal permeability.

As a protein cargo to be conjugated with MTD 1067, we chose GHRP-6, des(1-3)IGF-I, and PDGF-BB, which are useful pharmaceutical and cosmeceutical components (Table 1). GHRP-6 is a synthetic peptide that specifically stimulates growth hormone secretion in several animal species and humans^39,40^. GHRP-6 is known as hexapeptide-2 (HP-2) in the cosmetic field, which is currently used as an active ingredient for skin brightening, exploiting its melanogenesis inhibitory activity as an antagonist of α-MSH. GHRP-6 is also used as a stimulator of collagen synthesis based on its myogenic stimulating activity^41^. IGF-I is involved in various cellular functions, including the regulation of metabolic processes, cellular differentiation, transformation, and apoptosis suppression, and is responsible for normal cell growth and health maintenance^42–44^. IGF-I has recently been used as an active ingredient for hair growth and facial management in the cosmeceutical field^45^. Des(1-3)IGF-I is a truncated variant of human IGF-I lacking the 3 amino acids (Gly-Pro-Glu) at the N-terminus. Des(1-3)IGF-I was shown to be approximately ten fold more potent than IGF-I in stimulating hypertrophy and proliferation of cultured cells, usually resulting in much reduced binding to IGF-binding proteins^46^. PDGF is a growth factor that regulates cell growth and division. It is a homodimer or heterodimer consisting of one of four isoforms (A, B, C, and D) of the PDGF growth factor family^47^. PDGF plays a significant role in blood vessel formation and growth, mitogenesis, and chemotactic and angiogenic properties^48^. PDGF-BB is an important mitogen and a potent motility source for human dermal fibroblast (HDF) chemotaxis^49^, thus activating the HDF production of matrix proteins, including fibronectin^50^, collagen^51,52^, and hyaluronic acid^53^.

All the MTD 1067-conjugated cargoes tested in this study retained their inherent biological activities, such as anti-melanogenesis, collagen synthesis, or cell proliferation effects, which are identical or improved compared to the original cargoes (Figs. 3, 4, and 5). In the skin penetration activity of MTD 1067-conjugated peptide and recombinant proteins (Fig. 6), it is notable that MTD 1067 exhibited different penetration efficiency in the permeability of cargoes of various sizes in the artificial skin penetration test, with a superior enhancement effect on the permeability of larger proteins, such as PDGF-BB, compared to GHRP-6 peptides consisted of six amino acids. A few CPPs, such as arginine-rich IMT-P8 and small cyclic diketopiperazine (DKP) peptide, were previously reported on their skin penetration capacity. The IMT-P8-conjugated green fluorescent protein, GFP, and proapoptotic peptide, KLA, were shown to be internalized into the mouse skin after topical application^54^. The DKP-conjugated diclofenac, a non-steroidal anti-inflammatory drug, showed enhanced penetration into human epidermis^55^. Compared to the previously reported CPPs, our data suggested that MTD 1067 could be applicable to various protein cargoes in the wider ranges of MW and organization without detrimental effect on the intrinsic activity of cargoes. Furthermore, all the MTD 1067-conjugated peptide and protein cargoes tested in this study did not negatively affect cell viability without detectable cytotoxic effects up to a concentration of 1.898 mg/mL (GHRP-6, 1,000 μM) or 10 μg/mL (des(1-3)IGF-I, 1.17 μM; PDGF-BB, 0.37 μM) in normal human dermal fibroblast adult cells (HDFa) (Fig. 7). Taken together, our results strongly support the high potential of the hydrophobic peptide MTD 1067 as a promising vehicle for the transdermal delivery of functional protein cargoes of various sizes and structures in the development of advanced cosmetics and pharmaceuticals. The extended repertories of new CPPs, including MTD 1067, will help broaden the applicability of protein-peptide conjugates with the claimed bioactivity and safety as novel ingredients in cosmeceutical industry.

## Materials and methods

### Cell lines, cell culture, and reagents

The murine myeloid cell line FDC-P1, mouse embryonic fibroblast cell line NIH 3T3, murine melanoma cell line B16F10, normal human dermal fibroblast adult cell (HDFa), and HaCaT cells were obtained from the American Type Culture Collection (ATCC, USA). HDFa and NIH 3T3 cells were grown in DMEM (Dulbecco’s modified Eagle’s Media; Invitrogen, USA) medium supplemented with 10% fetal bovine serum (FBS; Hyclone, USA). FDC-P1 cells were grown in DMEM medium supplemented with 10% fetal bovine serum (FBS; Hyclone, USA) and 5 ng/mL mIL-3. All cells were maintained at 37 °C in a humidified 5% CO_2_ incubator for 24 h. EpidermFT was purchased from MatTek (MatTek Corporation, USA). Amino acids used for peptide synthesis were purchased from GL Biochem (Shanghai, China). The reagents and organic solvents were purchased from Sigma-Aldrich. All chemicals used were of analytical grade.

### Chemical synthesis and purification of GHRP-6 and MTD 1067-conjugated GHRP-6 peptides

GHRP-6 and MTD-GHRP-6 were synthesized using the solid-phase peptide synthesis (SPPS) method (Supplementary Fig. S1a, Supplementary Table S1). The synthesized peptides were cleaved from the resin, purified using reverse-phase column chromatography, and lyophilized using a freeze-dryer. The purified GHRP-6 and MTD-GHRP-6 peptides were analyzed by reverse-phase (RP)-HPLC using a 3.5 μm Waters Xbridge BEH 300 C18 column (4.6 cm × 250 mm) with a Waters liquid chromatography system. Elution was performed with a gradient formed by mixing solutions A [0.1% (v/v) TFA in water] and B [0.08% (v/v) TFA in 80% acetonitrile] as follows: 20–60% B (0–25 min), 60–100% B (25–35 min), 100–20% B (35–50 min), 20% B (50–60 min). The flow rate was maintained at 1 mL/min and the column effluent was monitored at 220 nm. Fluorescein isothiocyanate (FITC)-labeled forms of GHRP-6 and MTD-GHRP-6 were prepared by adding FITC during the SPPS synthesis process^56,57^.

### Expression and purification of recombinant MTD 1067-conjugated des(1-3)IGF-I and PDGF-BB proteins

The genes encoding des(1-3)IGF-I and PDGF-BB were synthesized based on sequence information of des(1-3)IGF-I and PDGF-BB proteins (Table 1, Supplementary Table S1) with codon optimization for *E. coli* overexpression via Genscript (GenScript, NJ, USA). The codon optimized des(1-3)IGF-I and PDGF-BB genes were subcloned in pUC57 vector, renerating pUC57-des(1-3)IGF-I and pUC-PDGF-BB, respectively. For the expression of recombinant des(1-3)IGF-I and MTD-des(1-3)IGF-I proteins, the vectors pET11a-des(1-3)IGF-I and pET11a-MTD-des(1-3)IGF-I (Supplementary Fig. S2a) were constructed by subcloning the PCR-amplified DNA fragments of des(1-3)IGF-I and MTD-des(1-3)IGF-I, amplified from pUC57-des(1-3)IGF-I using the primer sets listed in Fig. S2a, into pET11a (Merck KGaA, Germany) and transformed into *E. coli* BL21 (DE3). For expression of recombinant PDGF-BB and MTD-PDGF-BB proteins, the vectors pET17b-PDGF-BB and pET17b-MTD-PDGF-BB (Supplementary Fig. S2b) were constructed by subcloning the PCR-amplified DNA fragments of PDGF-BB and MTD-PDGF-BB, amplified from pUC57- PDGF using the primer sets listed in Fig. S2b into pET17b (Merck KGaA, Germany) and transformed into *E. coli* BL21 (DE3). Overexpression of recombinant proteins (des(1-3)IGF-I, MTD-des(1-3)IGF-I, PDGF-BB, and MTD-PDGF-BB) was induced by adding 1 mmol/L isopropyl-beta-d-thiogalacto-pyranoside (IPTG) to Luria–Bertani (LB) medium and additionally cultivated at 200 rpm at 37 °C for 3 h. After harvesting *E. coli* cells by centrifugation (7000 rpm, 10 min, 4 °C), the lysis buffer (50 mmol/L Tris–HCl, pH 8.0, 10 mmol/L EDTA) was added to the cell pellets (20 mL per g cells), which were disrupted using ultrasonication.

For purification of des(1-3)IGF-I and MTD-des(1-3)IGF-I proteins, expressed as insoluble forms, inclusion bodies (IBs) were obtained by centrifugation (8000 rpm, 20 min, 4 °C), suspended in a solubilizing buffer solution (6 mol/L guanidine, 100 mmol/L Tris–HCl, 1 mmol/L EDTA, pH 9.0), and stirred at room temperature for 6 h. The solubilized samples were added to the final 0.2 mg/mL in refolding buffer (100 mmol/L Tris–HCl, 30% ethanol, 0.5 mmol/L β-mercaptoethanol, pH 9.0) and incubated at 4 °C for 48 h. The refolded des(1-3)IGF-I and MTD-des(1-3)IGF-I proteins were purified using SP Sepharose chromatography and size-exclusion chromatography (Cytiva, USA). For purification of PDGF-BB and MTD-PDGF-BB, separated IBs were suspended in a solubilizing buffer solution (6 mol/L guanidine, 10 mmol/L dithiothreitols (DTT), pH 8.0) and stirred at room temperature for 4 h. The solubilized samples were added to the final 0.2 mg/mL in refolding buffer (100 mmol/L Tris–HCl, 0.4 mmol/L GSH, 0.01 mmol/L GSSG, pH 8.0) and incubated at room temperature for 24 h. The refolded PDGF-BB and MTD-PDGF-BB proteins were purified using CM Sepharose chromatography (Cytiva, USA). The purified recombinant proteins were concentrated and stored in a cryogenic refrigerator at − 80 °C. To check the purity of the purified proteins, sodium dodecyl sulfate–polyacrylamide gel electrophoresis (SDS-PAGE) was performed under reduced and non-reduced conditions. Purity was analyzed by measuring the intensity of all bands on the SDS-PAGE gel using an image quantification program (Totallab Quant software).

### Western blot analysis

After SDS-PAGE, the proteins were transferred to the PVDF membrane (300 mA, 1 h), which was blocked using 5% skim milk for 1 h. In the reaction with the primary antibodies, anti-human IGF1 monoclonal antibody (R&D Systems, USA) and anti-human PDGF-BB polyclonal antibody (R&D Systems, USA), respectively, were used for incubation 16 h at 4 °C. As the secondary antibodies, anti-mouse IgG-alkaline phosphatase antibody (Merck KGaA, Germany) and anti-goat/sheep IgG-alkaline phosphatase antibody (Merck KGaA, Germany) were added for the subsequent reaction (1 h, room temperature) for the detection of IGF1 and PDGF-BB, respectively. After then, AP conjugate substrate (Bio-Rad Laboratories, USA) was added and incubated for 10 min for color development.

### Cell proliferation activity assay

The cell proliferation activities of des(1-3)IGF-I and MTD-des(1-3)IGF-I were determined by crystal violet staining of HDFa cells. The cultured HDFa cells were inoculated in a 12 well plate at a density of 2 × 10^4^ cells/well, cultured for 24 h, and replaced with serum-free DMEM. Serially diluted proteins were added to the HDFa cells in each well and cultured for 72 h. After incubation, the cells were stained with 0.5% crystal violet and analyzed through microscopic observation. The cell proliferation activity of des(1-3)IGF-I and MTD-des(1-3)IGF-I was also measured using FDC-P1 cells and HaCaT cells, which were cultured using DMEM medium supplemented with 10% FBS and 5 ng/mL mIL-3 (R&D systems, USA). The cultured cells were inoculated in a 96-well plate at 5 × 10^3^ cells/well and cultivated for 24 h. Protein samples (100 μL), serially diluted with 5% FBS DMEM medium, were added to each well and incubated at 37 °C in a humidified 5% CO_2_ incubator for 48 h. Ten microliters of WST-1 solution (Roche, Switzerland) were added to each well and incubated for 4 h, and the absorbance was measured at 450 nm using a microplate reader (Spectra Max, Molecular Devices, USA).

The cell proliferation activity of PDGF-BB and MTD-PDGF-BB was measured using NIH 3T3 cells, which were cultured in DMEM medium supplemented with 10% Bovine Calf Serum (BCS; Hyclone, USA) in a 96-well plate at 5 × 10^3^ cells/well for 24 h. 100 μL of the protein sample, serially diluted with serum-free DMEM medium, was added to each well and cultured at 37 °C for 48 h. After removing the cell culture medium, 100 μL of a mixed solution of 5 mg/mL MTT and serum-free DMEM at a ratio of 1:10 was added to each well. After 4 h of incubation in a CO_2_ incubator for 4 h, the reaction solution was removed, and 100 μL of DMSO was added to each well. Formazans were sufficiently dissolved in DMSO using a plate shaker to develop a purple color, which was measured at 570 nm using a microplate reader (Spectra Max, Molecular Devices, USA).

### Analysis of stimulation activity of collagen synthesis

The ability of peptides and proteins to stimulate collagen synthesis was evaluated using HDFa cells, which were cultured in DMEM medium supplemented with 10% fetal bovine serum (FBS; Hyclone, USA) at 37 °C for 24 h. The cultured cells were inoculated in a 24 well plate at 1 × 10^4^ cells/well and cultured for 24 h. The proteins were diluted in serum-free DMEM medium, added to the HDFa cells at concentrations of 10, 50, and 250 ng/mL, and cultured at 37 °C for 24 h. The amount of collagen in the HDFa culture supernatant was measured using a procollagen type I C-peptide (PIP) EIA kit (Takara, Japan).

### Analysis of melanogenesis inhibition activity

Murine melanoma B16F10 cells were plated on dishes at 1 × 10^4^ cells/mL, incubated with GHRP-6 and MTD-GHRP-6 to concentrations of 100, 200, and 400 μM for 2 h, and treated with 100 nM α-MSH for 48 h. After washing twice with PBS, the cells were detached by incubation in Trypsin/EDTA, cell pellet was recovered by centrifugation at 10,000 rpm for 5 min. For measuring intracellular melanin amount, the cell pellets were dissolved in 500 µL of 1 N NaOH, incubated at 60 °C for 1 h. Melanin content in the samples was determined by measurement of the absorbance at 490 nm. The bicinchoninic acid (BCA) protein assay was used for protein content (Pierce Biotechnology, USA). The melanogenesis activity (%) was calculated by the formula below, in which the control is the cell culture treated with α-MSH in the absence of GHRP-6 and MTD-GHRP-6:

$$Melanogenesis \left(\%\right)=\frac{Absorbance \; of \;sample}{Absorbance \; of \;control} \times 100$$

### Cell cytotoxicity test

HDFa cells were seeded in a 96-well plate to 5 × 10^3^ cells/well and cultivated in 0.1 mL DMEM medium supplemented with 10% fetal bovine serum for 24 h. GHRP-6 and MTD-GHRP-6 peptides were serially diluted with serum-free DMEM to concentrations of 0, 10, 50, 100, 500, and 1000 μM, and des(1-3)IGF-I, MTD-des(1-3)IGF-I, PDGF-BB, and MTD-PDGF-BB proteins were serially diluted with serum-free DMEM to concentrations of 0, 0.001, 0.01, 0.1, 1, and 10 μg/mL, and the diluted peptides and proteins were added to each well. Cytotoxicity was confirmed using a lactate dehydrogenase (LDH) assay. To perform the LDH assay, 50 μL of cell culture solution was transferred to a new 96-well plate and 50 μL of LDH substrate mix solution (Promega, USA) was added to each well. After incubation at room temperature for 30 min, the reaction was stopped and absorbance was measured at 490 nm using a microplate reader (Spectra Max, Molecular Devices, USA).

Cell viability was confirmed using the MTT assay reagent (Merck KGaA, Germany). For the MTT assay, the culture medium was removed, and 100 μL of a mixed solution of 1:10 ratio of 5 mg/mL MTT solution and serum-free DMEM was added. The reaction was carried out at 37 °C for 4 h. After completion of the reaction, the reaction solution was removed and 100 μL of DMSO was added to each well. The absorbance of the plate was measured at 570 nm using a microplate reader (SpectraMax, Molecular Devices, USA).

### Confocal laser scanning microscope

To evaluate skin permeability using confocal microscopy, des(1-3)IGF-I and MTD-des(1-3)IGF-I were labeled with fluorescein isothiocyanate (FITC), and PDGF-BB and MTD-PDGF-BB were labeled with rhodamine. The labeling reaction was carried out with the molar ratio of protein: FITC (or rhodamine) as 1:10 at 4 °C for 2 h. Excess FITC and rhodamine were removed using a PD-10 desalting column (GE Healthcare, USA). After adjusting the concentrations of FITC-labeled GHRP-6, MTD-GHRP-6, des(1-3)IGF-I, MTD-des(1-3)IGF-I, PDGF-BB, and MTD-PDGF-BB to 0.2 mg/mL, respectively, each labeled solution was applied to the artificial skin (EpiDermFT™, MatTek, USA). The EpidermFT™ skin model consists of normal human epidermal keratinocytes (NHEK) and normal human dermal fibroblasts (NHDF) provided in Costar Snapwell™ single well tissue culture plate inserts with 1.2 cm-diameter and 1.0 cm^2^-surface area in the 6 well plate. The enclosed artificial skin tissues were transferred to 2.5 mL culture medium, provided by the supplier, and incubated at 37 °C, 5% CO_2_ for 16 h. 40 μL of each solution of labeled peptide and proteins was applied to the artificial skin, and the permeation reaction was performed in an incubator at 32 °C, 5% CO_2_ for 24 h. After 24 h of incubation, the artificial skin surface was washed three times with PBS, separated, and placed in an embedding cassette. The artificial skin was fixed in 10% formalin solution at 4 °C for 24 h. Frozen sections were collected after the sucrose infiltration. The degree of penetration of the treated samples into the artificial skin was observed and photographed using a confocal microscope (LSM 700, Carl Zeiss, Germany). The fluorescence intensity per equivalent area of the dermal layer was calculated for each confocal image. Quantification analysis using fluorescence intensity was calculated based on the fluorescence intensity of confocal microscopy images. Three confocal microscopy images were obtained per each artificial skin, and the fluorescence intensity values were extracted from five areas of the same size per dermis layer in the microscopy images. The relative intensity was calculated by comparing the signals between the samples with and without MTD conjugation.

### Statistical analysis

The statistical significance of each data according to the experimental group was verified through Student's t-test, and when the p-value < 0.05 or less, it was considered that there was a statistically significant difference.

## Supplementary Information

Supplementary Information.

## Data Availability

The DNA sequences encoding Des(1-3)IGF-I, MTD-des(1-3)IGF-I, PDGF-BB, and MTD-PDGF-BB were deposited under GenBank accession numbers, BankIt2569064 Des(1-3)IGF-I ON156849, BankIt2569065 MTD-des(1-3)IGF-ION156850, BankIt2569068 PDGFB ON156851, and BankIt2569071 MTD-PDGFB ON156852, respectively, at NCBI (https://www.ncbi.nlm.nih.gov/genbank/).
