# Supplementary material for: Efficient transdermal delivery of functional protein cargoes by a hydrophobic peptide MTD 1067
Source: Sci Rep. 2022 Jun 27;12:10853. doi: 10.1038/s41598-022-14463-9 (PMC9237094; doi:10.1038/s41598-022-14463-9)
Supplement: Supplementary file 1 — Supplementary Information. [file 41598_2022_14463_MOESM1_ESM.pdf]

Supplementary Information

Efficient transdermal delivery of functional protein cargoes by a hydrophobic peptide MTD 1067

Hee Je Shin[1,2,#], Sun Uk Bak[1], Ha Na La[1], Jin Sun Kang[1], Hwa Hyun Lee[1], Hyo Jung Eom[1], Byung Kyu Lee[1], Hyun Ah Kang[2, #]

[1]ProCell R&D Center, ProcellTherapeutics. Inc., #1009 Ace-Twin Tower II, 273, Digital-ro, Guro-gu, Seoul 08381, Korea.

[2]Department of Life Science, College of Natural Science, Chung-Ang University, Seoul 156-756, Korea.

[#]Correspondence

Hee Je Shin (email: hjshin@procellrx.co.kr)

Hyun Ah Kang (email: hyunkang@cau.ac.kr)

Contents

Supplementary Tables S1

Supplementary Figures S1-S4

Table S1. Amino acid sequences of the synthesized MTD-GHRP-6 peptide and DNA sequences of the *E. coli*-codon optimized genes for MTD-des(1-3)IGF1 and PDGF-BB proteins

**MTD-Cargoes**	**Peptide & DNA sequences***[2]
MTD-GHRP-6 Peptide*[1]	HW(D)AWF(D)KGG**AAVAPAAARM**
MTD-des(1-3)IGF1 DNA	ATGACCCTGTGTGGTGCGGAACTGGTGGATGCTCTGCAATTCGTCTGTG<br>GTGACCGTGGCTTCTACTTCAATAAACCGACCGGCTATGGTAGCTCTAGT<br>CGTCGCGCGCCGCAGACCGGTATTGTGGATGAATGCTGTTTTCGTAGCT<br>GTGACCTGCGTCGTCTGGAAATGTATTGTGCCCCGCTGAAACCGGCGAA<br>ATCGGCGGCG**GCGGTGGCGCCGGCGGCGGCGCGCATGTAA**
MTD-PDGF-BB DNA	ATGTCCCTGGGCAGCCTGACCATCGCAGAACCGGCAATGATCGCAGAAT<br>GCAAGACCCGCACCGAAGTGTTTGAAATCTCCCGTCGCCTGATTGATCG<br>TACCAACGCGAATTTTCTGGTCTGGCCGCCGTGCGTGGAAGTTCAGCGC<br>TGTAGCGGCTGCTGTAACAATCGTAACGTTCAATGCCGTCCGACGCAGG<br>TCCAACTGCGTCCGGTCCAGGTGCGCAAAATTGAAATCGTGCGTAAAAA<br>GCCGATCTTCAAAAAGGCCACCGTTACGCTGGAAGACCATCTGGCGTGT<br>AAATGCGAAACGGTTGCGGCGGCTCGTCCGGTGACC**GCGGTGGCGCC**<br>**GGCGGCGGCGCGCATGTAA**

*[1] W(D) and F(D) in MTD-GHRP-6 peptide sequence are meaning D-amino acids.

*[2] Amino acid and DNA sequences of MTD 1067 are indicated in bold.

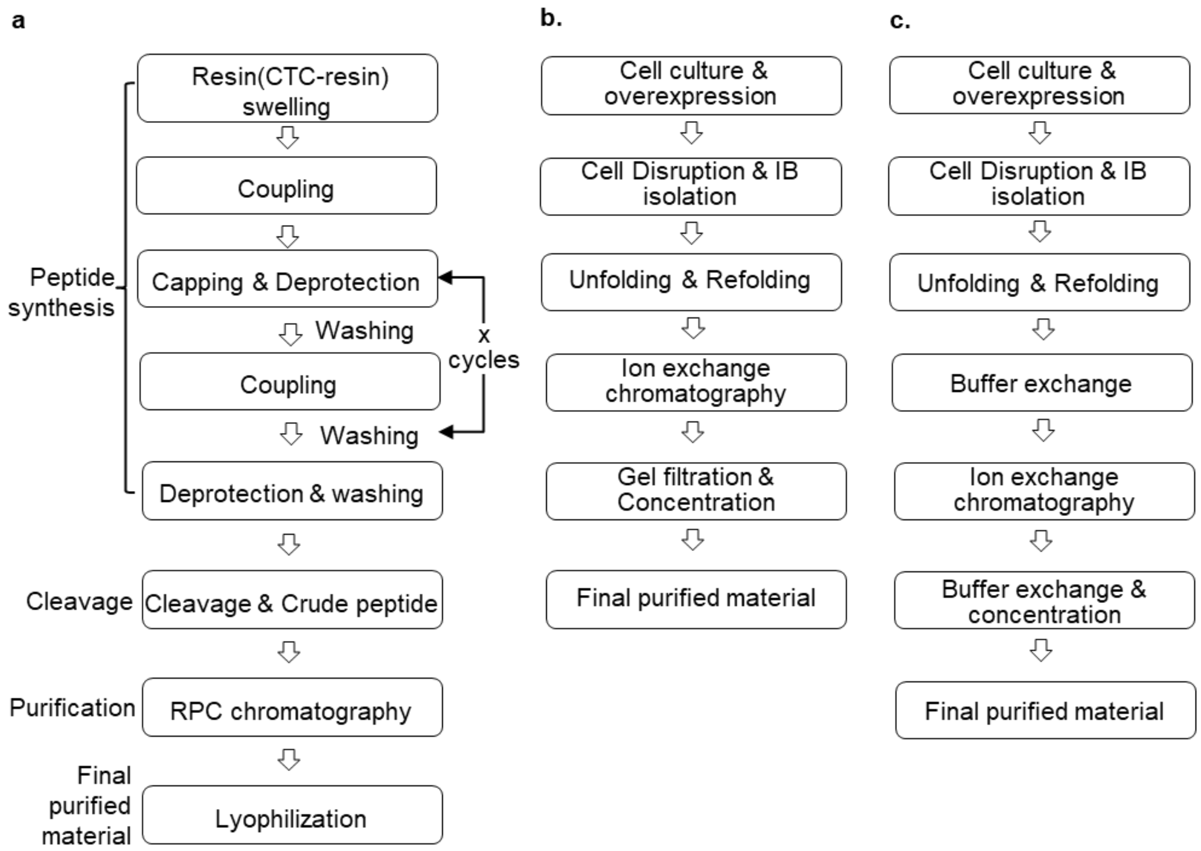

Figure S1. Peptide synthesis and recombinant protein production. Flow diagrams of the synthesis and purification of GHRP-6 and MTD- GHRP-6 (a), the purification of des(1-3)IGF-I and MTD-des(1-3)IGF-I (b), and the purification of PDGF-BB and MTD-PDGF-BB purification (c).

a.

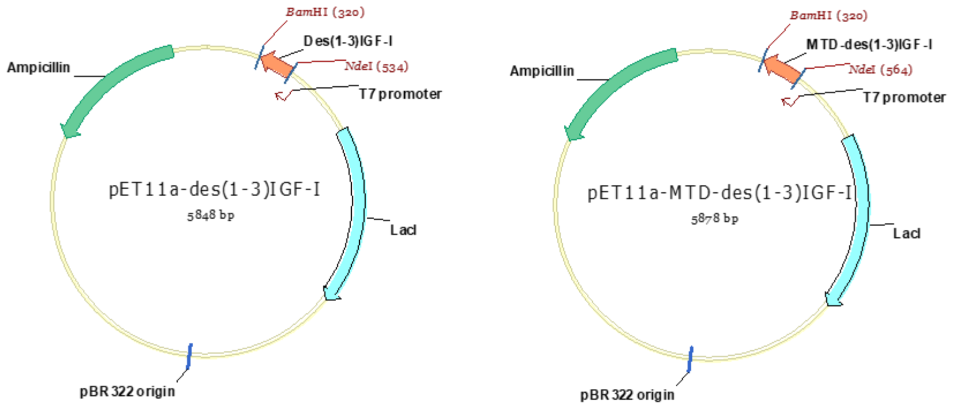

Des(1-3)IGF-I primer set

Forward primer	5'-TATATCATATGACCCTGTGTGGTGCG-3'
Reverse primer	5'-CGCGGATCCTTACATGCGCGCCGC-3'

MTD-des(1-3)IGF-I primer set

Forward primer	5'-ATATCATATGACCCTGTGTGGTGCG-3'
Reverse primer	5'CAAAGGATCCTTACATGCGCGCCGCCGCCGGCGCCACCGCCGCCGCCGATTT CGCCGGTTT-3'

b.

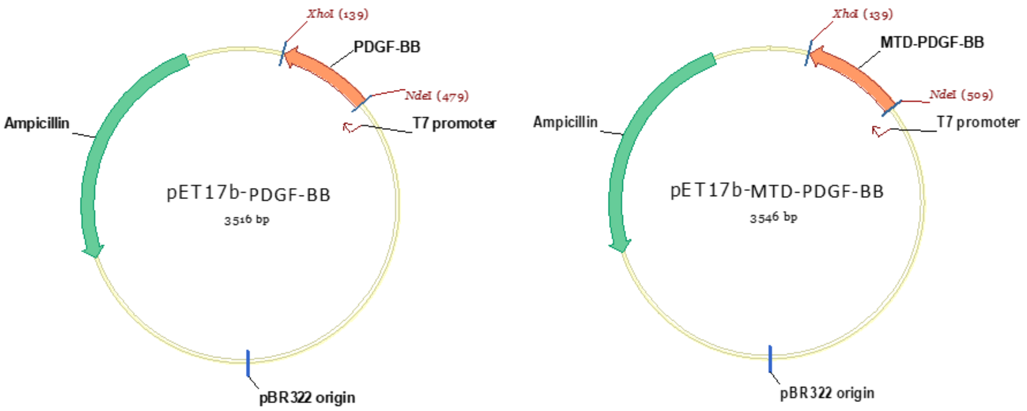

PDGF-BB primer set

Forward primer	5'-TATATCATATGTCCCTGGGCAGCCTGAC-3'
Reverse primer	5'-CAAACTCGAGTCAGGTCACCGGACGAGCCG-3'

MTD-PDGF-BB primer set

Forward primer	5'-TATATCATATGTCCCTGGGCAGCCTGAC-3'
Reverse primer	5'CAAACTCGAGTCACATGCGCGCCGCCGCCGGCGCCACCGCCGCGGTCAC CGGACGAGC-3'

Figure S2. Vector maps for the expression of recombinant proteins and the primer sets used for amplification of the target genes. Des(1-3)IGF-I and MTD-des(1-3)IGF-I (a), PDGF-BB and MTD-PDGF-BB (b).

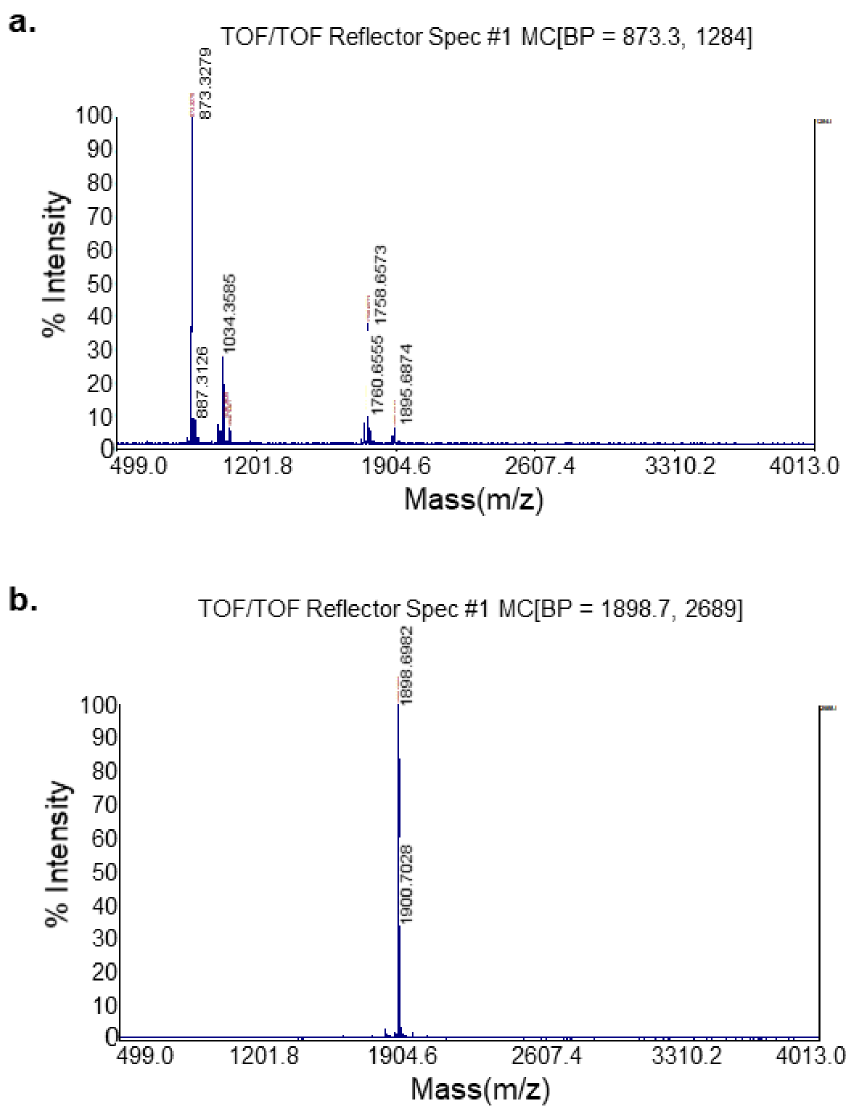

Figure S3. Mass analysis of purified GHRP-6 (a) and MTD-GHRP-6 (b) peptides. The mass spectrometry of the peptide was analyzed using MALDI-TOFTOF (Matrix-Assisted Laser Desorption Ionization Mass Spectrometer, MALDI-TOFTOF 5800 System, AB SCIEX, USA), and was performed under reflector mode, positive mode, and 20KV accelerating voltage.

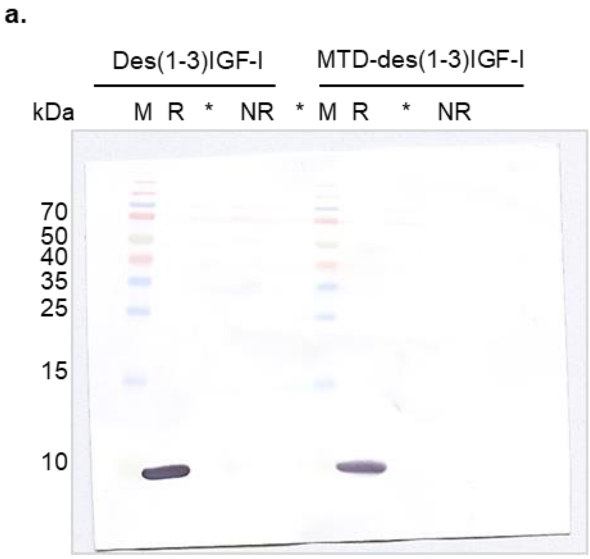

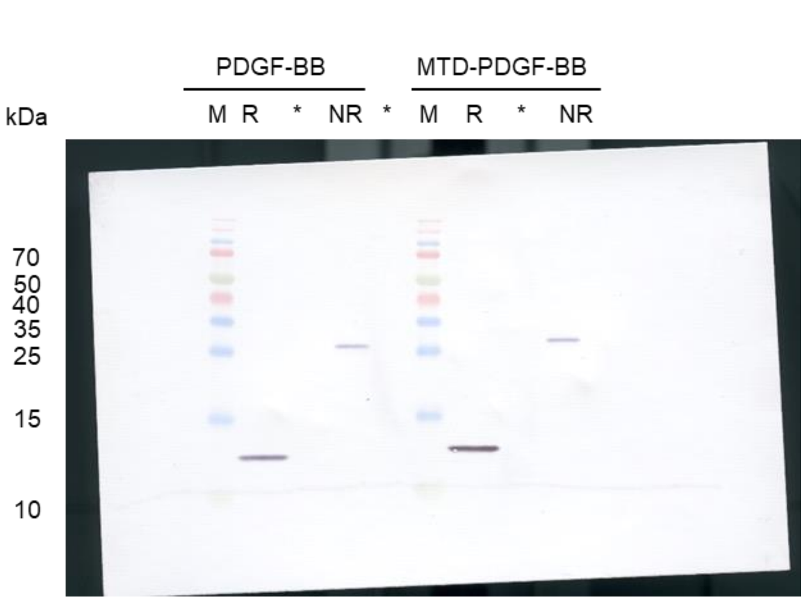

Figure S4. Raw data of western blotting of recombinant proteins with and without MTD conjugation. (a) Des(1-3)IGF-I and MTD-des(1-3)IGF-I proteins, (b) PDGF-BB and MTD-PDGF-BB proteins. M, molecular weight marker; R, reduced form; NR, non-reduced form; *, blank lane without sample loading. To avoid interference by β-mercaptoethanol present in the R samples during electrophoresis, the protein samples were not loaded in the lane between R and NR samples was not loaded.
